# Peri-saccadic compression to two locations in a two-target choice saccade task

**DOI:** 10.3389/fnsys.2015.00135

**Published:** 2015-10-06

**Authors:** Markus Lappe, Fred H. Hamker

**Affiliations:** ^1^Department of Psychology, Institute for Psychology & Otto Creutzfeldt Center for Cognitive and Behavioral Neuroscience, University of MuensterMuenster, Germany; ^2^Department of Computer Science, Chemnitz University of TechnologyChemnitz, Germany

**Keywords:** saccade, spatial perception, decision making, efference copy, oculomotor system

## Abstract

When visual stimuli are presented at the onset of a saccadic eye movement they are seen compressed onto the target location of the saccade. This peri-saccadic compression is believed to result from internal feedback pathways between oculomotor and visual areas of the brain. This feedback enhances vision around the saccade target at the expense of localization ability in other regions of the visual field. Although saccades can be targeted at only one object at a time, often multiple potential targets are available in a visual scene, and the oculomotor system has to choose which target to look at. If two targets are available, preparatory activity builds-up at both target locations in oculomotor maps. Here we show that, in this situation, two foci of compression develop, independent of which of the two targets is eventually chosen for the saccade. Our results suggest that theories that use oculomotor feedback as efference copy signals for upcoming eye movements should take the possibility into account that multiple feedback signals from potential targets may occur in parallel before the execution of a saccade.

## 1. Introduction

Saccadic gaze shifts during the viewing of a scene result from a complex interplay between target selection, allocation of attention, movement planning, and movement initiation. This interplay is orchestrated by the communication between many areas of the brain. Even when only a single target is present, as in many typical experiments on saccades, a number of processes have to be completed in the latency period before saccade initiation: The target is sensed and represented in visuomotor areas, attention is allocated to the target location (Kowler et al., 1995; Deubel and Schneider, 1996; Bisley and Goldberg, 2003), preparatory activity begins to build-up in motor regions (Glimcher and Sparks, 1992; Munoz and Wurtz, 1995; Hanes and Schall, 1996), fixation is released (Fischer and Boch, 1983; Dorris and Munoz, 1995), and finally the saccade is initiated by a burst of activity of motor neurons (Bruce and Goldberg, 1985; Munoz and Wurtz, 1995; Hanes and Schall, 1996). The bulk of these activities takes place within the last 50 ms or so before saccade initiation. During this time period the visual system allows for a number of transient localization errors or omissions in visual perception, presumably because it is preoccupied with the upcoming gaze shift and mainly interested in the new visual input that will soon be obtained after the saccade (Dodge, 1900; Matin and Pearce, 1965; Bischof and Kramer, 1968; Bridgeman et al., 1975; Burr et al., 1994; Ross et al., 1997; Yarrow et al., 2001; Morrone et al., 2005). These phenomena are often related to the insurance of perceptual continuity across the saccade, and are believed to involve an efference copy signal or corollary discharge of the saccade command (Honda, 1989; Duhamel et al., 1992; Bridgeman et al., 1994; Deubel et al., 1998; Ross et al., 2001; Sommer and Wurtz, 2002; Pola, 2004; Binda et al., 2007; Melcher and Colby, 2008; Cavanagh et al., 2010; Hamker et al., 2011; Ziesche and Hamker, 2011, 2014; Zirnsak and Moore, 2014).

Among the transient errors around saccade execution is the peri-saccadic compression of the perceived location of flashed stimuli onto the saccade target location (Bischof and Kramer, 1968; Morrone et al., 1997; Ross et al., 1997; Lappe et al., 2000; Kaiser and Lappe, 2004). By supporting simulations of a neuro-computational model, we have proposed that peri-saccadic compression is generated by the feedback of the saccadic targeting signal from oculomotor structures into visual areas (Hamker et al., 2008). Such feedback enhances the visual processing in the target area, giving rise to attentional benefits for visual discrimination. This benefit comes at the expense of distorting the activity profile of the visuo-spatial maps (Tolias et al., 2001; Zirnsak et al., 2014) such that stimuli are drawn toward the saccade target. If the driving force behind peri-saccadic compression is the target selection or motor planning signal, the model predicts compression to a single location—the saccade target.

However, target selection and motor planning become more complicated when multiple potential targets are present. In this case, a decision has to made which target to look at. Before that decision is made, however, preparatory oculomotor signals are generated for all available targets in the superior colliculus (Glimcher and Sparks, 1992; Basso and Wurtz, 1997), the lateral intraparietal area (LIP) (Platt and Glimcher, 1997; Shadlen and Newsome, 2001) and the frontal eye field (Schall and Hanes, 1993; Lee and Keller, 2008). The activity at multiple target locations is kept until close to the onset of the saccade (Thompson et al., 2005; Thomas and Pare, 2007; Kim and Basso, 2008). If feedback from these areas is the driving force behind peri-saccadic compression one might expect that compression will be generated to multiple foci. Thus, we can ask the question about the origin of the proposed feedback signal.

## 2. Methods

### 2.1. Participants

Four subjects (2 female, 2 male, between 25 and 44 years old) participated in the study. All had normal or corrected to normal vision. All subjects gave informed consent. All procedures were in accord with the guidelines of the institutional ethics committee and conformed to the Declaration of Helsinki. One subject was an author of this study. Two subjects were completely naive to the objective of the investigation.

### 2.2. General setup and eye movement recording

The experiment took place in a dimly lit room. The head of the subjects was supported by a chin rest during the experimental session. Stimuli were presented on a 19”-Monitor (Samsung 95P plus) with a visible display size of 35.6 × 28.4 cm. The viewing distance of 40 cm to the screen resulted in a visual field of 48 × 39°. The full-screen images had a resolution of 1280 by 1024 pixel presented with a frame rate of 144 Hz. Movements of the eyes were recorded with a video based eye tracker (EyeLink II, SR Research, Inc.) at a sampling rate of 500 Hz.

### 2.3. Procedure

Each trial began with the appearance of a small white fixation point (size 0.4 by 0.4°; luminance 28 cd m^−2^) on a red background (luminance 11 cd m^−2^) at position (-8, 0) deg with respect to the screen center. The fixation point was presented for a randomized time interval between 500 and 1500 ms. Then it disappeared, and either one or two saccade targets of the same color and size as the fixation point were presented, and stayed on for the rest of the trial. These target locations were (+10, +6) and (+10, −6) with respect to the screen center. The three conditions (upper target only, lower target only, both targets) were presented in pseudo-random order and with equal probability. The subject was instructed to make a saccade as quickly as possible to the target, in case a single target was present, or to a target of his or her choice, if two targets were present. Between 50 and 350 ms after target onset a small green stimulus (size 0.6 by 0.6°; luminance 33 cd m^−2^) was flashed for one video frame (7 ms) at a pseudo-randomly chosen location of one out of four possible locations: (+4, −4), (+4, +4), (+16, −8), and (+16, +8). The subject was instructed to report the apparent position of the flash after the saccade with a mouse pointer that appeared 500 ms later. In case the subject did not perceive the flash the instruction was to click on the right edge of the screen. The mouse click started the next trial.

A single recording session lasted between 100 and 200 trials. At least four sessions were recorded for each subject.

Subjects S1, S2, and S4 also participated in a further condition in which single saccade targets were presented in blocks of trials. This condition served as a control that the intermixing between single and double trials in the main experiment did not change mislocalization patterns for the case of a single target. Since the results of the blocked and the randomized single target trials were very similar both data sets were pooled for the final analysis.

### 2.4. Data analysis

Data were analyzed in Mathematica (Wolfram Research). The onset of a saccade was defined as the first of three sequential eye position samples with a velocity above 22°/s and an acceleration above 3800°/s^2^. Dot presentation times with respect to saccade onset were calculated on a trial by trial basis from the saccade latency and the presentation times of the dot. Trials in which the latency was not between 100 and 300 ms or in which a small saccade (less than 9° horizontal component) occurred after target onset were omitted from further analysis. This concerned 30% of trials in the main experiment and 60% of trials in the blocked single target conditions, largely because subject S1 had very unstable fixation. Consequently, this subject performed more sessions in order to achieve a number of usable trials similar to the other subjects. Trials in which the subject did not perceive the flashed dot were also omitted from analysis. This occurred in 5% of trials. The total number of trials that were included in the analysis of the main experiment for the four subjects were: 455, 455, 615, and 374. The number of trials in the blocked single target conditions for the three participating subjects was: 184, 281, and 261.

## 3. Results

**Figure 1:**
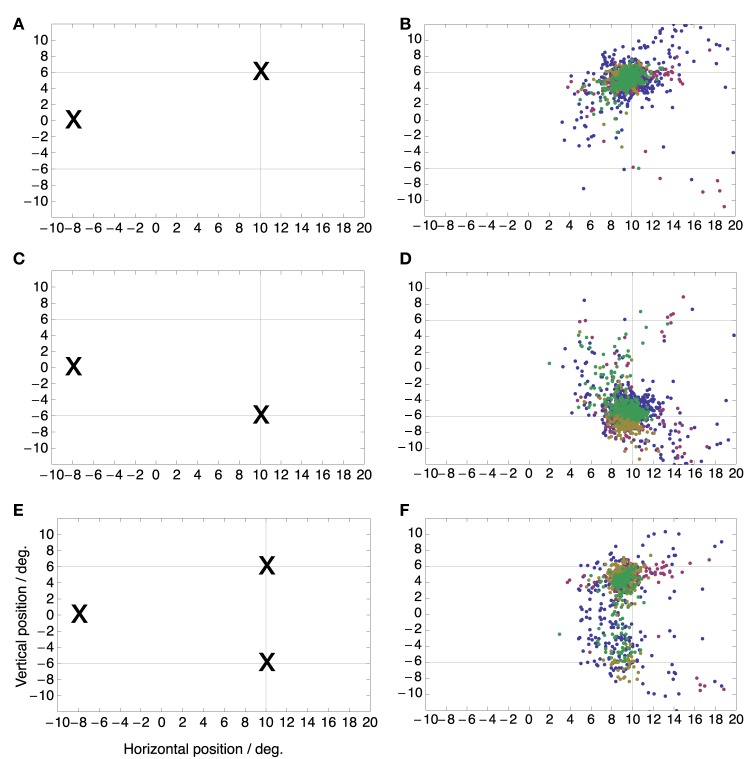
**Saccade target locations (Left) and distributions of saccade landing positions (Right) for the three saccade target conditions**. In the single target conditions **(A–D)** the initial fixation point was at (−8, 0) deg while the saccade target was either at (+10, +6) deg **(A,B)** or at (+10, −6) deg **(C,D)**. In the double target condition **(E,F)**, the initial fixation point was again at (−8, 0) deg but both targets were shown simultaneously and the subject was free to choose any one of the targets for making the saccade. Color refers to the different subjects.

Figure 1 shows the three target conditions and the resulting distributions of saccade landing positions color coded by subject. The saccade targets appeared 10° to the right of the screen center and 6° above or below the midline. In the two single target conditions the majority of saccades are directed to the target. On average the saccades were slightly hypometric, which is normal for saccades of this size. Mean saccadic endpoints were (9.4°, 4.8°) for the upper target location and (9.1°, 6.0°) for the lower target location. In the double target condition, saccade landing positions were distributed between the two target locations with a bias toward the upper location which was consistently chosen more often than the lower target location in three of the four subjects. A small percentage of saccades landed between the two targets. Median latencies for the three conditions were 161 ms (upper target), 175 ms (lower target), and 169 ms (both targets). Median latencies of the individual subjects were 153, 162, 182, and 171 ms. Straightness or curvature of saccade trajectories did not differ between single and double target conditions.

**Figure 2:**
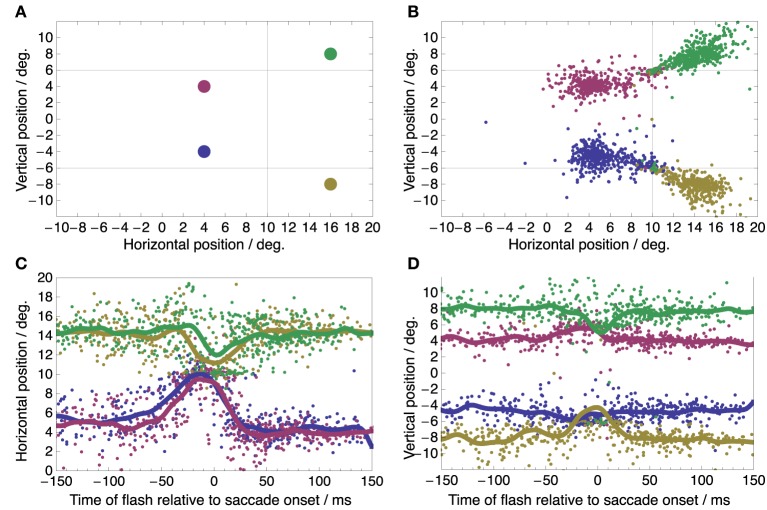
**Spatial distribution and time course of perceived flash locations. (A)** True locations of the four flashes. In any single trial, one of these flashes was presented before, during, or after the saccade. **(B)** Perceived flash locations pooled from all subjects, conditions, and trials show compression toward the saccade targets at (+10, +6) and (+10, −6) deg (marked by the intersection of the thin horizontal and vertical lines). **(C,D)** Time course of perceived flash locations showing the familiar temporal pattern of peri-saccadic compression (**C**: Horizontal component of perceived location; **D**: vertical component of perceived location).

While subjects performed these saccades a small green dot was flashed for 7 ms at a one out of four possible locations on the screen, randomized between trials. These locations were (+4, −4), (+4, +4), (+16, −8), and (+16, +8) deg relative to the screen center (Figure 2A). They were thus arranged such that two locations fell along the saccade vector to each target, one located between fixation point and target and the other located beyond the target. These flashes served to probe the peri-saccadic compression to either target. Subjects had to report the perceived location of the flash with a mouse pointer after saccade completion.

Figure 2B shows the spatial distributions of perceived locations of the four flashes pooled over all conditions. Most perceptual reports are near the true flash locations but some are clearly compressed toward the saccade targets. Such compression is known to occur time-locked to the onset of the saccade. Figures 2C,D show the reported horizontal (C) and vertical (D) flash locations as a function of flash time relative to saccade onset. The horizontal and vertical components were plotted to allow an easy comparison to earlier studies of peri-saccadic compression with respect to time course (Morrone et al., 1997; Ross et al., 1997; Lappe et al., 2000; Kaiser and Lappe, 2004). The curves for perceived location show the typical features of peri-saccadic compression: apparent horizontal location is compressed toward the saccade target position at 10° in the time range between -50 and 30 ms with a peak at saccade onset (Morrone et al., 1997; Ross et al., 1997); compression starts and peaks a few milliseconds later for flashes beyond the saccade target (green and yellow curves in Figure 2C) compared to flashes between saccade target and fixation point (red and blue curves) (Lappe et al., 2000; Kaiser and Lappe, 2004); vertical compression toward the target positions at +6 and -6° is more pronounced for flash locations beyond the saccade target (green and yellow curves in Figure 2D) (Kaiser and Lappe, 2004). Thus, we conclude that our experimental conditions sufficed to induce clear peri-saccadic compression.

**Figure 3:**
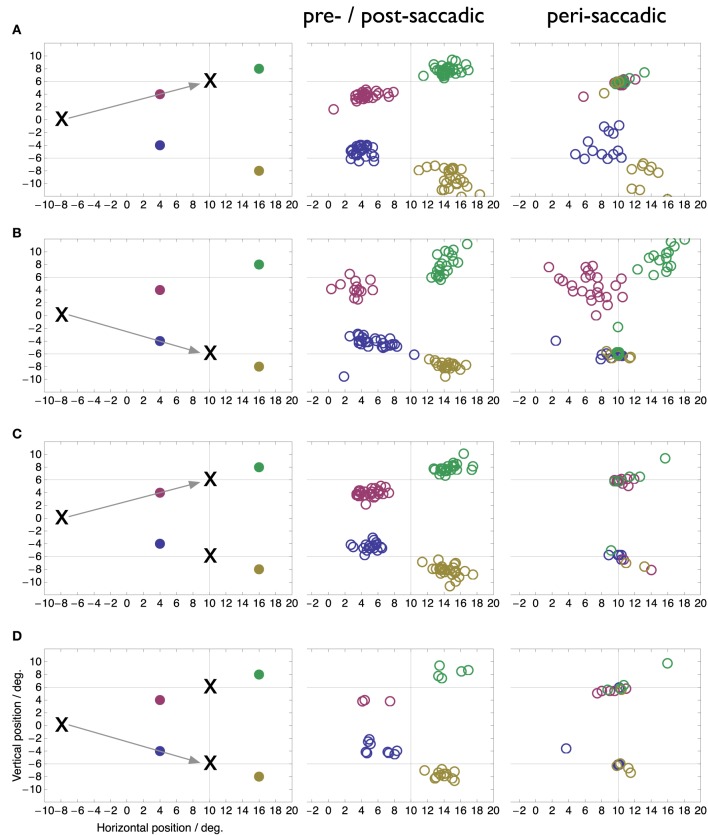
**Perceived locations of flashes presented more than 100 ms before or after saccade onset (pre-/post-saccadic) or around the onset of the saccade (peri-saccadic, ±10 ms around saccade onset in A–C, ±20 ms in D)**. True flash locations as well as experimental conditions are shown in the left colum. **(A)** Saccade to single upper target. **(B)** Saccade to single lower target. **(C)** Saccade to upper of two simultaneous targets. **(D)** Saccade to upper of two simultaneous targets.

The goal of our study is to determine the focus of peri-saccadic compression in double target conditions when a motor choice has to be performed. We therefore compared the perceived locations of peri-saccadic flashes in the double target condition with those in the singe target conditions. We split the data into a peri-saccadic time period of ±10 ms around saccade onset, when the compression is maximal, and a pre-/post-saccadic baseline period without compression which includes all data that were collected more than 100 ms before or after saccade onset. In the case of a saccade to the downward target in the two target condition the peri-saccadic time range was increased to ±20 ms since this condition contained comparatively few saccades. Figure 3 shows perceived peri-saccadic and pre-/post-saccadic locations in four conditions: (A) saccades to a single upper target, (B) saccades to a single lower target, (C) saccades to the upper target when two targets were presented, and (D) saccades to the lower target when two targets were presented. In the latter two cases, the stimulation was the same and the performed saccade was entirely up to the choice of the subject in that particular trial. To select saccades that were directed to either the upper or the lower target a circle of 3° radius was drawn around the median landing locations in the single target locations and only saccades that landed within this circle were submitted to the analysis.

Figure 3 illustrates several new findings obtained with the double-target choice task. First, compression occurs toward both target locations not only toward the one chosen for the saccade. This is clearly visible in the peri-saccadic plots in rows C and D as the peri-saccadic flashes were perceived closer to one of the two targets than the pre-/post-saccadic flashes (Mann-Whitney *U*-test on the median distance of the perceived position to the nearer of the two targets, *p* < 0.05). In comparison, in the single target condition flashes near the target location (red and green in A, blue and yellow in B) are fully compressed onto the target while flashes further apart show more modest mislocalization. Second, compression of each flash is mostly directed toward the nearest of the two targets. However, in some trials the flash appeared fully compressed even onto the farther of the two targets. For example, some of the green and red data points in row C appear at the bottom target location. Moreover, in these trials the saccade went to the upper target. Thus, compression to the farther target occurred even when that target was not the landing point of the saccade. Third, compression was directed to the target locations, not the landing points of the saccade. As can be seen in Figure 1 the saccade landing points show a fair amount of variability and our selection of saccades from within a 3° radius around each target included most of that variability. The compression, however, is fully focused on the target locations.

**Figure 4:**
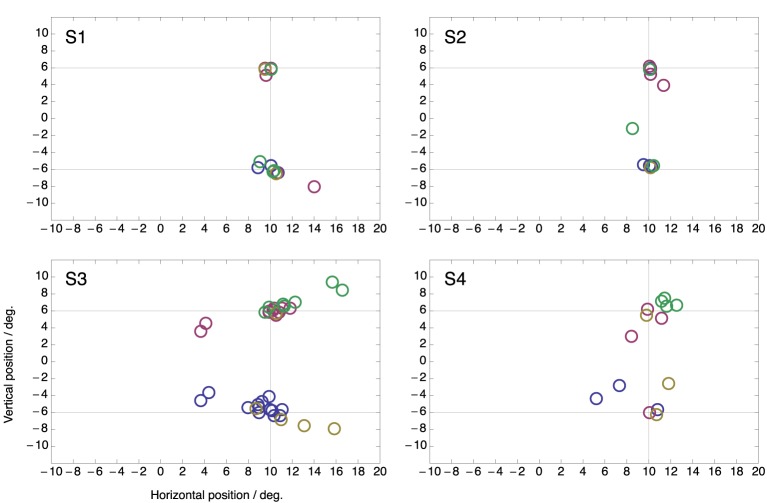
**Perceived positions of all peri-saccadic flashes (±15 ms from saccade onset) in the two-target condition for the four subjects**. Each panel presents all trials as if the saccade were made toward the upper target. Hence, for trials in which the saccade was made to the lower target saccade data as well as perceived positions were flipped along the horizontal meridian.

Figure 4 shows peri-saccadic perceived positions for each individual subject in the two-target condition. In order to draw all data in the same panel, in trials in which the saccade was made to the lower target, saccade data as well as perceived positions were flipped along the horizontal meridian. Thus, the figure presents all trials as if the saccade were made toward the upper target. For each subject, all flashes within ±15 ms around saccade onset were used. Moreover, all saccades were included, even those that landed not close to a target. The figure clearly shows that the compression onto both targets was present in each individual subject. Moreover, because in these plots the saccade is never to the lower target, the figure also shows that the mislocalization toward the not-chosen target occurs in each subject.

**Figure 5:**
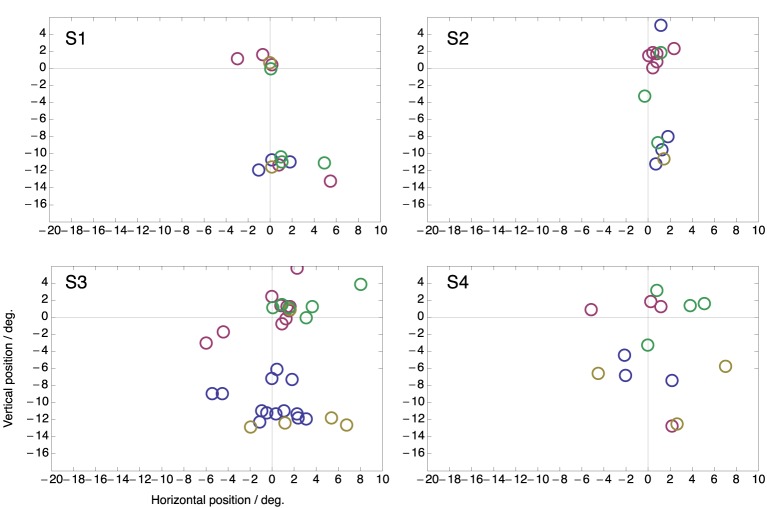
**Same data as in Figure 4 but plotted as perceived position relative to the end point of the saccade**.

Figure 5 shows the same data but now plotted as perceived position with respect to the end point of each saccade. There is considerably more spread than when the data is plotted with respect to the targets (Figure 4). This confirms that compression was directed to the targets rather than to the end point of the saccade that was made. Note that, if compression were directed toward the end point of the saccade then perceived compression should have clustered at position (0, 0) in this plot.

## 4. Discussion

Our results show that the focus point of peri-saccadic compression does not depend on the executed saccade. If two target locations compete for the execution of a saccade both become foci of compression. However, since the compression occurs only within a few milliseconds around saccade onset, and not during fixation long before or after the saccade, some process associated with saccade generation is likely to be involved. At present the most comprehensive account to peri-saccadic compression has been given by a neuro-computational model which predicts a distortion of population activity in extrastriate visual maps by feedback signals from oculomotor areas (Hamker et al., 2008, 2011). Previous simulations have assumed only a single saccade target. However, this model could account for multiple foci of compression if one assumes several activity hills in oculomotor areas that feed back to visual areas and each distorts a part of the visual representation. There is indeed much electrophysiological evidence for this. When multiple saccade targets are present preparatory oculomotor activity in the build-up neurons of the superior colliculus (Glimcher and Sparks, 1992; Basso and Wurtz, 1997), in the parietal cortex (Platt and Glimcher, 1997; Shadlen and Newsome, 2001) and the frontal eye field (Schall and Hanes, 1993; Lee and Keller, 2008) is generated at each target location in parallel.

Rather than preparatory oculomotor activity, one may think that the visual appearance of the targets drives compression. Because compression is so strongly focused on the target location, this explanation has some appeal. However, the compression occurs time-locked to the saccade, and is absent when the subject keeps fixating (Morrone et al., 1997; Ross et al., 1997). Moreover, studies with anti-saccades and saccadic adaptation have shown that the compression is linked to the saccade, not the target (Awater and Lappe, 2004; Awater et al., 2005). However, since the planning of a saccade involves multiple stages of transformation form visual to motor signals, some intermediate representations could be the origin of the proposed feedback signal. This conceptual framework relates well to recent research in visual attention where it has been a matter of debate if attention can be split simultaneously to multiple non-contiguous locations (Jans et al., 2010). Among others (Dubois et al., 2009) recently reported evidence for a split of spatial attention to two non-contiguous locations target shapes that were intermitted by distractors. Such a split of attention can be well explained by a computational model that continuously feeds back the activity of visuomovement cells in the frontal eye field (Zirnsak et al., 2011).

Compression toward the visual targets rather than the end point of the saccade may seem in conflict with previous studies that showed compression toward the saccade end point. These studies used anti-saccades (Awater and Lappe, 2004), saccadic adaptation (Awater et al., 2005), or the Müller-Lyer illusion (Matziridi et al., 2013) to separate the end point from the visual target. We believe that these findings, too, can be reconciled by considering a feedback signal from intermediate stages of the oculomotor transformation. For example, saccadic adaptation has been shown to affect perceived target location (Zimmermann and Lappe, 2010). The Müller-Lyer illusion, likewise, affects both perception and saccades (Bruno et al., 2010). Anti-saccades involve the establishment of a motor plan in the FEF to saccade toward the side opposite from the target. Hence, in all these cases there must be an intermediate stage that represents the motor target of the upcoming saccade and that may serve as the feedback signal. In the present study, since the representation of both targets in the intermediate stages is kept until close to saccade onset compression shows two foci.

Zimmermann et al. (2014) have recently suggested that a process associated with saccade generation is not needed to explain compression since backwards masking induces a similar pattern of spatial compression without saccade execution. However, compression only occurs if prior to the flash an anchor stimulus is presented. This will likely, similar as saccade targets, activate oculomotor areas which in turn feed back to visual areas as proposed by neuro-computational models of attention (Zirnsak et al., 2011). Further, a mask reduces flash visibility akin to saccadic suppression. Reduced visibility is a component of peri-saccadic compression (Georg et al., 2008; Hamker et al., 2008) which may explain why Atsma et al. (2014) did not observe compression with abruptly canceled saccades, as in their study the stimulus has not been masked and may not be sufficiently affected by saccadic suppression. Finally, masking erases immediate visual representations and forces the system to retrieve the flash location from visual memory. Such a reliance on memory representation has also been proposed as part of the mechanisms of peri-saccadic compression (Awater and Lappe, 2006; Hamker et al., 2008).

Concluding, corollary discharge is typically understood as a copy of the motor command that is fed back and used elsewhere in the brain as an anticipatory signal. For example feedback from the superior colliculus can inform other parts of the brain about the amplitude, direction, and timing of an upcoming saccade. However, our data suggest that in conditions of multiple targets the corollary discharge signal may not always be singular. Perhaps one must take into account the possibility that the colliculus, as well as other oculomotor areas, may generate multiple corollary discharge signals from preparatory saccade activity. The oculomotor feedback model (Hamker et al., 2008) offers a solution to our and previous findings because the onset of stimuli triggers preparatory activity at multiple locations which produces distortion of the population activity before the saccade is initiated and even when no saccade is executed. This distortion, is stronger with low stimulus visibility and generates compression toward the target or a stimulus location. If two potential targets are available, feedback from the preparatory signals at each location distorts the population activity such that two foci of compression become established.

### Conflict of interest statement

The authors declare that the research was conducted in the absence of any commercial or financial relationships that could be construed as a potential conflict of interest.
